# Radiocarbon Dating and Wood Density Chronologies of Mangrove Trees in Arid Western Australia

**DOI:** 10.1371/journal.pone.0080116

**Published:** 2013-11-12

**Authors:** Nadia S. Santini, Quan Hua, Nele Schmitz, Catherine E. Lovelock

**Affiliations:** 1 The School of Biological Sciences, The University of Queensland, St Lucia, Brisbane, Queensland, Australia; 2 Institute for Environmental Research, Australian Nuclear Science and Technology Organisation, Lucas Heights, New South Wales, Australia; 3 Plant Biology and Nature Management, Vrije Universiteit Brussel, Brussels, Belgium; DOE Pacific Northwest National Laboratory, United States of America

## Abstract

Mangrove trees tend to be larger and mangrove communities more diverse in tropical latitudes, particularly where there is high rainfall. Variation in the structure, growth and productivity of mangrove forests over climatic gradients suggests they are sensitive to variations in climate, but evidence of changes in the structure and growth of mangrove trees in response to climatic variation is scarce. Bomb-pulse radiocarbon dating provides accurate dates of recent wood formation and tree age of tropical and subtropical tree species. Here, we used radiocarbon techniques combined with X-ray densitometry to develop a wood density chronology for the mangrove *Avicennia marina* in the Exmouth Gulf, Western Australia (WA). We tested whether wood density chronologies of *A. marina* were sensitive to variation in the Pacific Decadal Oscillation Index, which reflects temperature fluctuations in the Pacific Ocean and is linked to the instrumental rainfall record in north WA. We also determined growth rates in mangrove trees from the Exmouth Gulf, WA. We found that seaward fringing *A. marina* trees (∼10 cm diameter) were 48±1 to 89±23 years old (mean ± 1**σ**) and that their growth rates ranged from 4.08±2.36 to 5.30±3.33 mm/yr (mean ±1**σ**). The wood density of our studied mangrove trees decreased with increases in the Pacific Decadal Oscillation Index. Future predicted drying of the region will likely lead to further reductions in wood density and their associated growth rates in mangrove forests in the region.

## Introduction

Mangrove forests are an important component of tropical and subtropical coastal ecosystems. In the arid zone of Australia, the Exmouth Gulf, Western Australia (WA), mangroves support an important fishery and biodiversity [1]. The stature and productivity of mangrove forests is correlated with latitude [2], [3] with trees tending to be larger and communities more diverse in tropical latitudes. In arid regions, diversity of mangrove forests is lower than in wetter regions and maximum tree height is also reduced [4], [5]. Arid regions are characterized by high evapotranspiration / rainfall ratios (>10) due to low rainfall and high temperatures [6]. Rainfall is likely to be an important factor determining the variation in productivity and diversity of mangrove trees in arid regions because it reduces the salinity of soil porewater (which limits water uptake and growth of mangrove trees) through dilution and recharge of groundwater and increases in soil moisture [7]. Additionally, growth of leaf bearing twigs has been observed to be enhanced after large rain-bearing cyclonic events, possibly due to delivery of nutrients and to increased availability of freshwater that can enhance metabolic rates [8]. Projections of rainfall with climate change indicate an increasing drying with less rainfall and higher temperatures in WA and also in parts of central Australia [9], [10]. However, examples of impacts of climate change in estuarine ecosystems are scarce [11], [12].

In the arid north west of Australia, at our study site at the Exmouth Gulf, climatic patterns are complex. High rainfall (>600 mm/yr) occurs in the austral summer associated with tropical cyclones, but in some years rainfall can also be associated with southern low-pressure climatic systems during the winter months [13]. The Pacific Decadal Oscillation Index (PDO), which reflects temperature fluctuations in the Pacific Ocean for long periods of time (20 to 30 years), has also been linked to rainfall in central and southern Australia, although the mechanisms underlying the connection between PDO and rainfall in Australia are not clearly resolved [13]. Here we assessed the adequacy of the PDO as a rainfall proxy in the Exmouth Gulf in order to increase the temporal scale of our analysis because reliable rainfall records from the Australian Bureau of Meteorology are relatively recent (from 1966), coincident with economic development of this remote region of WA.

Tree rings have been widely used to infer past climate and establish the response of vegetation to climate and other factors, but the use of ring formation in wood to determine sensitivity of trees to environmental change in mangroves and other tropical tree species can be problematic. In many tropical species annual growth rings are not produced or there is low confidence in the regular timing of tree-ring formation [14]. Examination of wood from the widespread mangrove species *Avicennia marina* (Acanthaceae) and from the Rhizophoraceae, indicates that changes in xylem vessel density throughout the life of trees can reflect changing environmental conditions [15], [16], [17], [18]. These studies suggest that variation in freshwater inputs (rain, rivers, groundwater) may strongly influence the structure of the wood, including its density. Our previous work on *A. marina* suggests that variation in tree growth rates correlates with predictable changes in wood density, and therefore wood density may be used as a proxy for tree growth rates over time [19].

The development of highly accurate and precise radiocarbon dating techniques that use the atomic bomb pulse of radiocarbon in the atmosphere in the 1960s to date recent organic materials including wood [20], [21] has offered advances in the examination of the response of tropical and subtropical trees [22], [23] to variation in climate. Here, we used radiocarbon techniques combined with X-ray densitometry (which allows analyses of continuous and high-resolution wood density profiles from tree stems over time [24], [25]) to develop a wood density chronology. We tested whether wood density chronologies of *A. marina* were sensitive to variation in the Pacific Decadal Oscillation Index, and determined growth rates in mangrove trees from the Exmouth Gulf, WA.

## Materials and Methods

### Site description and sample collection

The study site was located in Giralia Bay, in the east of the Exmouth Gulf, WA (22.4°S, 114.3°E). The climate of the site is warm and dry, with a mean air temperature of 25°C, a mean minimum temperature of 17°C, and a mean maximum temperature of 32°C, with peaks of up to 47°C in summer. The mean annual rainfall at the site is 250 mm/yr. In the region, cyclones bringing wind gusts in excess of 300 km/h and heavy rainfall (>600 mm/yr) occur every two to three years [26].

In October 2008, four mature stems of the widespread mangrove species *A. marina* were collected. The sampling was 7 months after tropical cyclone *Pancho*, which flooded the region in March 2008 [8]. In the region trees have multiple stems [27], we chose stems of ∼10 cm diameter and ∼3.3 m tall from the seaward edge of the forest for our study. The reasons were that: 1. Trees of this form are typical for the mangrove seaward fringing zone in the region and 2. The interior of larger stems is damaged by boring insects and bivalves (particularly shipworms), thereby preventing dating of larger girthed trees.

### Ethics statement

Our work was conducted under the permit numbers SW014933, SW013522, SW012157, SW010985, Department of Environment and Conservation, Western Australia. *Avicennia marina* is classified as a species of “least concern” according to the International Union for Conservation of Nature and Natural Resources (2013).

### Wood density determination

From the tree stems, we cut rectangular blocks of 4 mm ×4 mm x (maximum radius) of the stem with a bandsaw (CarbaTec, Australia), excluding pith and bark. We placed the wood blocks within glass containers with distilled water under vacuum for one day (sufficient time to fully saturate the samples) before scanning them with a compact micro CT scanner 1174 (SkyScan, Belgium). The wood blocks were sealed with plastic film to avoid desiccation and were exposed to X-rays (45 KV, 800 µA) for 30 minutes. We obtained four wood density profiles from each wood block (expressed as 256 grey values) from the CT scanner (resulting in a resolution of *ca*. 22 grey values for each mm of rectangular woody block). The wood density profiles were calibrated with the actual fresh (green) wood density values [28] from the wood blocks using Eqn 1, where *A_ij_* is the calculated wood density, *B_ij_* is the grey value along the stem (0 – 256) and *k* is a constant calculated as mean grey value times mean fresh wood density [29].
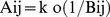
(1)


The high resolution of assessed wood density (*ca*. 22 values for each mm of rectangular woody bar) allowed us to perform wood density averages a) per mm of wood, and b) at dates established by radiocarbon dating. Our obtained wood density profiles are available at the International Tree-Ring Data Bank (access numbers *stem 1*: giralia1age, giralia1dist; *stem 2*: giralia2age, giralia2dist; *stem 3*: giralia3age, giralia3dist and *stem 4*: giralia4age, giralia4dist) [30]. To evaluate reliability of the wood density signal of the collected stems, we calculated the expressed population signal (EPS) from the wood density profiles (four wood density profiles per stem, *stem 1* – *stem 4*) with the package *detrendeR* from the software R [31], [32]. The EPS was determined by calculating the mean correlation between the wood density profiles [33]; a value of 0.85 of the EPS possible range (0 to 1) is considered the minimum to obtain a sufficiently replicated profile [34]. EPS of our wood density profiles was >0.85 (Supplementary material Figure S1, S2). Wood density profiles were detrended by calculating the ratio index of the wood density data to the associated values of the fitted smoothing spline using the package *detrendeR* from the software R [31], [32], [35]. The arithmetic means and standard deviation of wood density and detrended wood density were calculated for each stem (Supplementary material Figure S1, S2). Given the purpose of detrending data is to remove possible long-term trends due to tree aging, detrended wood density data were used for analysis in which the effect of tree aging had to be removed, while wood density data was used for analysis in which incorporation of tree aging was important. We specified in the Data Analysis section which density data (wood density or detrended wood density) were used for each analysis.

### Tree age and growth rate estimation

From the scanned rectangular blocks, we cut cross-sections along the radius of 1 mm length (resulting in 4 mm ×4 mm ×1 mm slices) at intervals of 4 – 20 mm to obtain 6 – 7 samples per stem. These samples were dated using bomb-pulse ^14^C techniques to estimate the growth history of each stem. The wood samples were pre-treated to extract alpha-cellulose using the method described in [36]. Alpha-cellulose was then combusted to CO_2_ and reduced to graphite [37] for ^14^C analyses using the STAR accelerator mass spectrometry (AMS) facility at the Australian Nuclear Science and Technology Organisation [38]. Measured ^14^C values were converted to calendar ages using the “Simple Sequence” deposition model of the OxCal calibration program based on chronological ordering (outer samples are younger than inner samples [39]), and a calibration data set for the Southern Hemisphere (SH) for the last 350 years consisting of the updated SH bomb radiocarbon data [20] and the SHCal04 data for the pre-bomb period [40].

### Tree growth rates

Growth rates were estimated for the mean calibrated ^14^C age of each wood slice from the gradient of a cubic spline fitted to the data [41]. Growth rates were determined as increments in stem circumference from the slope of a weighted linear regression through the plotted points and expressed as mm/yr.

### Data analyses

We used linear regression analyses to assess the relationship between rainfall and the Pacific Decadal Oscillation Index (PDO, obtained from [42], Supplementary material Figure S3) and sea level pressure and the PDO (obtained from [43], Supplementary material Figure S4). Rainfall data was obtained from the Australian Bureau of Meteorology [26], Learmonth Station (Number 5007, 32 km away from our site). The rainfall record was continuous between 1966 and 2008, but rainfall data were not available or were patchy over periods of time in which growth of our mangrove trees occurred (1919 – 1965). Regression analyses were also used to determine the relationship between wood density and growth rate (using dates established by ^14^C dating), wood density and distance from pith, wood density and time, detrended wood density and the PDO, detrended wood density and rainfall, growth rate and the PDO and growth rate and rainfall for the time in which rainfall data were available (1961, 1964, 1966 – 2008).

Finally, because of differences in the age of trees and to reflect major changes in the PDO (Supplementary material Figure S3, [44]), we grouped growth rate values within the following periods of time 1952 – 1976 (n = 12), 1977 – 1998 (n = 6) and 1999 – 2008 (n = 6). Differences in growth rates between these three periods of time were tested using Analysis of Variance (ANOVA). These tests were performed with the software Prism ver 5.0 (GraphPad Software, La Jolla, CA, USA).

## Results

**Figure 1:**
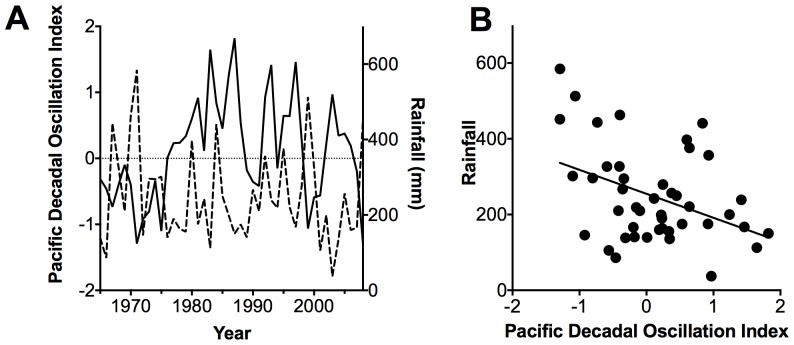
Relationship between annual rainfall and the Pacific Decadal Oscillation Index. Relationship between A) annual rainfall (dashed line) and the Pacific Decadal Oscillation Index (PDO, solid line) in the Exmouth Gulf, Western Australia between 1966 and 2008. B) The line represents the linear regression where Rainfall  = −63.2 PDO +255, *r^2^* = 0.16, *p* = 0.006, *n* = 44.

We found significant but variable trends between rainfall and the Pacific Decadal Oscillation Index (PDO) in the Exmouth Gulf, Western Australia (WA) for the period between 1966 – 2008. Rainfall decreased with increasing values of the PDO (*r^2^* = 0.16, *p* = 0.006, Figure 1A, B).

Our radiocarbon results indicated that *A. marina* mangroves in the Exmouth Gulf (∼10 cm diameter, fringe canopy of ∼3.3 m tall) were 48±1 to 89±23 years old (mean ±1**σ**) with an age range of 47 – 112 years old. Determination of age of most of our wood samples had an uncertainty of 1 to 2 years with the exception of stems dated before 1952 (Table 1). Growth rates of *A. marina* expressed as increments in stem circumference per year ranged from 4.08±2.36 mm/yr in the period between 1999 – 2008 to 5.30±3.33 mm/yr in the period between 1952 – 1976, with an overall mean growth rate of 4.84±2.66 mm/yr for the period between 1952 – 2008. Growth rates were not significantly different between the assessed periods of time (Table 2).

**Table 1:** Radiocarbon results, estimated tree ages and growth rates of *Avicennia marina*.

Stem	Distance from pith (mm)	^14^C Mean ±1σ (pMC)	Modelled calendar age Mean ±1σ (year AD) ^(a)^	Growth rate (mm/yr) ^(b)^
**1**	1	117.20±0.36	1960±1	7.18±0.66
	8	141.12±0.39	1964±1	12.03±4.65
	15	151.92±0.44	1970±1	8.47±2.57
	22	139.72±0.40	1975±1	9.64±1.82
	36	125.53±0.34	1982±1	5.65±2.26
	43	113.36±0.33	1994±1	5.66±0.06
	50	109.37±0.40	2000±2	5.71±0.94
	57(c)	-	2008	7.65±1.16
**2**	7	98.06±0.69	1936±17	3.42±0.76
	13	101.36±0.31	1947±9	4.85±0.17
	19	97.51±0.28	1951±6	6.69±0.89
	25	104.05±0.54	1957±1	6.38±0.19
	31	119.50±0.51	1961±1	3.49±1.12
	37	133.48±0.53	1976±1	2.36±0.14
	43	110.52±0.50	1999±2	1.83±0.45
	47(c)	-	2008	1.40±1.44
**3**	1	99.04±0.37	1919±23	1.79±4.82
	11	98.24±0.40	1936±18	1.94±2.15
	16	107.66±0.48	1958±1	2.13±3.62
	21	145.32±0.46	1971±1	2.56±1.08
	26	120.67±0.52	1985±1	3.11±1.51
	36	112.02±0.47	1996±2	3.52±2.20
	41(c)	-	2008	3.62±2.51
**4**	1	99.67±0.32	1945±10	2.79±3.62
	6	99.01±0.40	1952±2	2.80±0.99
	11	133.72±0.43	1964±1	3.01±1.10
	16	143.48±0.59	1972±1	3.66±1.66
	21	127.98±0.46	1980±1	4.66±1.07
	41	115.22±0.36	1991±2	5.38±1.72
	51(c)	-	2008	4.31±0.10

Values of ^14^C are shown in percent modern carbon (pMC). ^(a)^ Modelled calendar age in year AD at 68.2% confidence level. ^(b)^ Uncertainty associated with mean growth rate represents the maximum difference between growth rate estimates from the spline curve using upper and lower limits of the calibrated ^14^C age range compared with the mean ^14^C age [41] ^(c)^ Outermost samples were collected in 2008 and were not used for ^14^C analysis.

**Table 2:** Growth rates of *Avicennia marina* mangroves from the Exmouth Gulf, Western Australia.

Time	Growth rate (mm/yr)	*n*
**1952–1976**	5.30±3.33	12
**1977–1998**	4.66±1.11	6
**1999–2008**	4.08±2.36	6

Values are means ±1**σ**.

**Figure 2:**
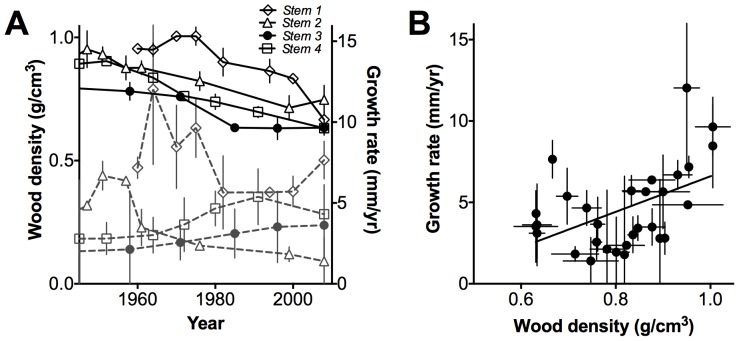
Relationship between growth rate and wood density of *Avicennia marina* collected in Giralia Bay, Western Australia. A) Relationship between growth rate (dashed grey line) and wood density (solid black line) from four stems (*stem 1* – *stem 4*) collected in Giralia Bay, Western Australia, points are at dates established using bomb-pulse dating, values are means ±1**σ**. B) The line represents the linear regression where: Growth rate  = 11.0 Wood density - 4.32, *r^2^* = 0.24, *p* = 0.006, *n* = 30. Points are means ±1**σ** at dates established using bomb-pulse dating.

Growth rates were positively correlated with wood density values in the fringing *A. marina* trees from the Exmouth Gulf, Western Australia (*r^2^* = 0.24, *p* = 0.006, Figure 2A, B). Results were significant when values of all the four stems were included in the analysis (Figure 2B) but analysis of individual stems of growth rates and wood density were not always significant.

**Figure 3:**
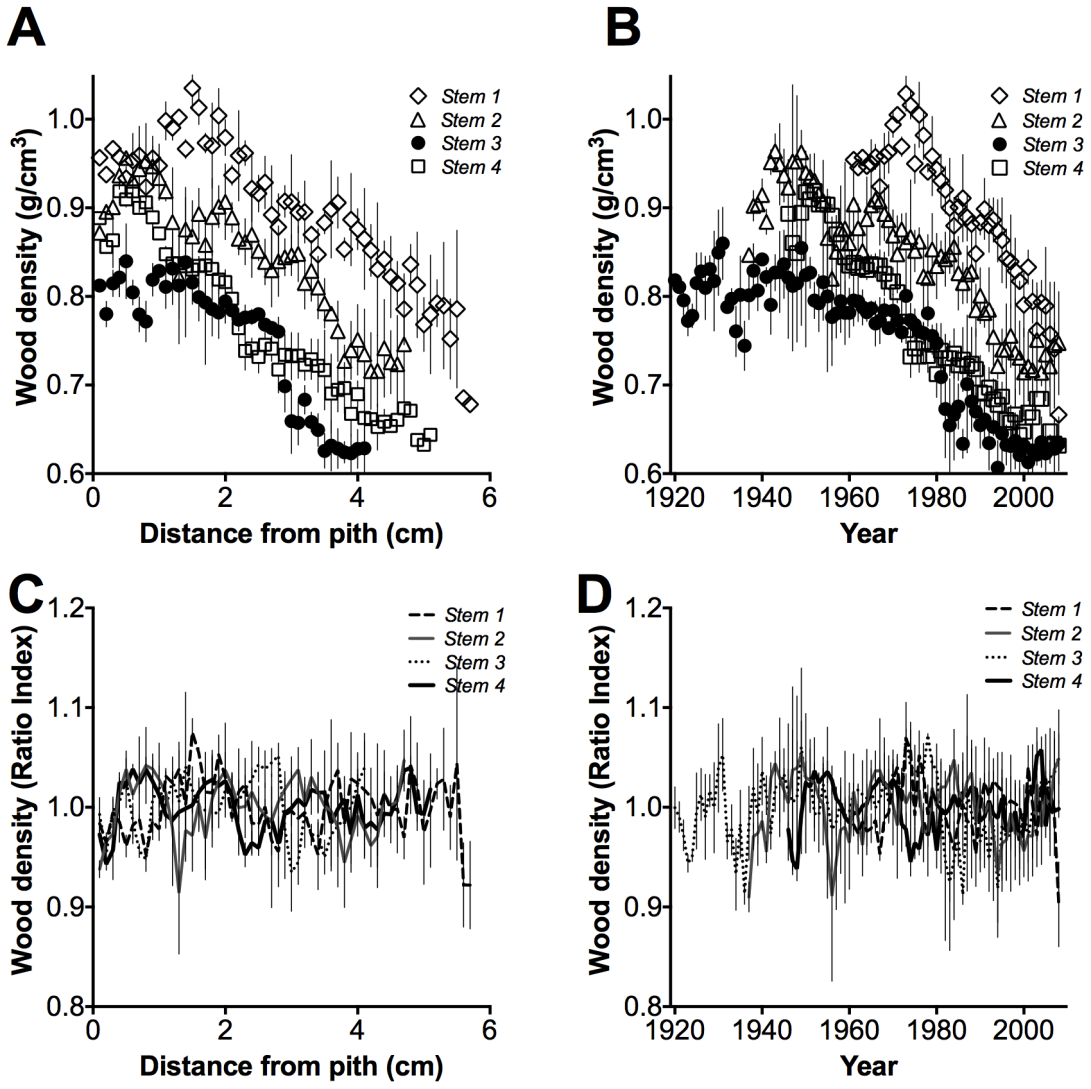
Relationship between wood density and distance from pith and wood density and time in *Avicennia marina*. Relationship between A) wood density and distance from pith, slopes between stems were significantly different (*p<0.0001*), the regression equations were: Wood density (*stem 1*)  = −4.3×10^−2^ distance from pith +1.01, *r^2^* = 0.78, *n* = 57; wood density (*stem 2*)  = −4.8×10^−2^ distance from pith +0.95, *r^2^* = 0.83, *n* = 47; wood density (*stem 3*)  = −5.5×10^−2^ distance from pith +0.86, *r^2^* = 0.78, *n* = 41; wood density (*stem 4*)  = −5.7×10^−2^ distance from pith +0.95, *r^2^* = 0.94, *n* = 51; *p*<0.0001 for all stems (*stem 1* – *stem 4*), vertical lines  = 1**σ**. Relationship between B) wood density and time, slopes between stems were significantly different (*p<0.0001*), the regression equations were: Wood density (*stem 1*)  = −5.07×10^−2^ time +10.9, *r^2^* = 0.78, *n* = 48; wood density (*stem 2*)  = −3.1×10^−2^ time +7.04, *r^2^* = 0.81, *n* = 72; wood density (*stem 3*)  = −2.5×10^−3^ time +5.75, *r^2^* = 0.76, *n* = 89; wood density (*stem 4*)  = −4.7×10^−3^ time +9.93, *r^2^* = 0.94, *n* = 63; *p*<0.0001 for all stems (*stem 1* – *stem 4*), vertical lines  = 1**σ**. C) Detrended wood density and distance from pith, vertical lines  = 1**σ** and D) Detrended wood density and time, vertical lines  = 1**σ**.

Linear regression analyses indicated that wood density decreased from pith to bark (*r^2^* = 0.78 – 0.94, *p*<0.0001) and with time in *A. marina* trees from the Exmouth Gulf (*r^2^* = 0.76 – 0.94, *p*<0.0001). Slopes of the linear regressions were significantly different among stems (*p*<0.0001) but consistently decreased from pith to bark and with time (1919–2008) for each stem (Figure 3A, B). However, detrended wood density did not significantly decrease from pith to bark or with time, but tended to vary over time (Figure 3C, D).

**Figure 4:**
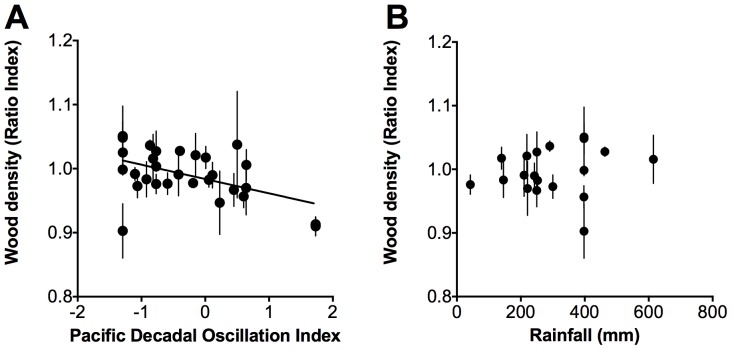
Relationship between detrended wood density and the Pacific Decadal Oscillation Index and detrended wood density and rainfall. Relationships between A) detrended wood density and the Pacific Decadal Oscillation Index (PDO) between 1919 and 2008. The line represents the linear regression where: Wood density (Ratio Index)  = −0.02 PDO +0.98, *r^2^* = 0.24, *p* = 0.03, *n* = 30 and B) detrended wood density and rainfall between 1961 – 2008. Points are means ±1σ.

**Figure 5:**
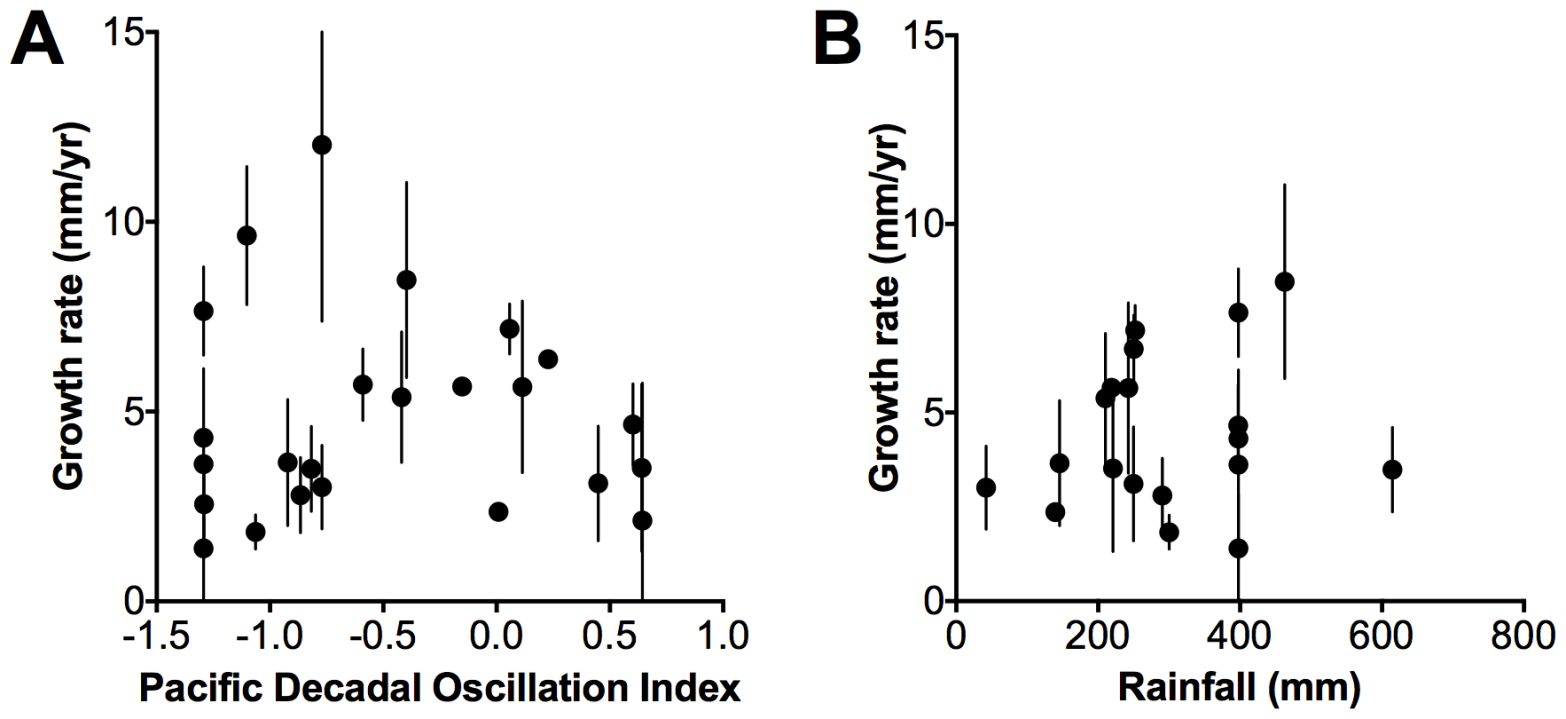
Relationship between growth rate and the Pacific Decadal Oscillation Index and growth rate and rainfall. Relationships between A) growth rate and the Pacific Decadal Oscillation Index (PDO) between 1952 and 2008 and B) growth rate and rainfall between 1961 – 2008. Points are means ±1**σ**.

We found a significant decrease in detrended wood density with increases in the PDO for 1919–2008 (*r^2^* = 0.24, *p = *0.03, Figure 4A). However, we found unclear trends between detrended wood density and rainfall for 1961 – 2008 (Figure 4B), growth rate and the PDO for 1952 – 2008 and growth rate and rainfall for 1961 – 2008 (Figure 5A, B).

## Discussion

Our results indicated that rainfall in the Exmouth region was correlated with the PDO, which in turn links to rainfall patterns in central and southern Australia [13]. Therefore, rainfall in the Exmouth region is associated with rainfall patterns of central and southern Australia.

The age of *A. marina* mangroves from the Exmouth Gulf was 48±1 to 89±23 years old (mean ±1**σ**) with an age range of 47 – 112 years old (Table 1). Uncertainty associated with age determination of wood samples was 1 – 2 years in recent wood samples (dated after 1952), but the uncertainty increased to 6 – 23 years in older samples (dated before 1952). The accuracy of radiocarbon dating and therefore in growth rates is higher for more recent samples (after 1952) because of significant increases in atmospheric ^14^C after 1955 resulted from atmospheric nuclear bomb tests [20].

The age of our sampled mangrove trees indicates that they had survived the devastating cyclone in 1998 (Cyclone *Vance*) that directly struck our study site [45] and also previous cyclonic events. Survival of seaward forests of *A. marina* in the Exmouth Gulf may be due to their resistant and flexible wood compared to other mangrove species [46], [47]. Some trees at the site reach 65 cm in circumference (C.E. Lovelock *unpublished data*), even in the shorter scrub forest (<1.5 m height) which occurs landward of the taller seaward fringe. Given the growth rates of trees at the site, these trees may be ∼160 years old (based on circumference increments of 4.08 mm/yr – the minimum growth rate for the last 60 years) or ∼120 years old (if circumference increments are 5.30 mm/yr – the maximum growth rate for the last 60 years). The estimated ages of larger girthed (although not taller) trees at this site are comparable to maximum ages of mangroves indicated by Alongi [48].

Growth rates of *A. marina* measured as increments in stem circumference per year exhibited an overall mean of 4.84±2.66 mm/yr (Table 2). These growth rates are comparable to stem growth rates of the same species observed in Gazi and Dabaso, Kenya [49]. Growth rates of *A. marina* exhibited higher values than growth rates for *Xylocarpus granatum* (0.62 – 2.51 mm/yr), *Bruguiera gymnorrhiza* (0 – 2.51 mm/yr), *Rhizophora mucronata* (0.94 mm/yr) and *Sonneratia alba* (0.31 – 1.25 mm/yr), all documented from mangrove trees of approximately 15 cm diameter from Micronesia [50].

We found growth rates were positively correlated with wood density values in the fringing *A. marina* trees from the Exmouth Gulf, Western Australia (Figure 2A, B). These results are in contrast to studies in other tree species where increasing density is often associated with low growth rates [51], [52], but are consistent with our previous work with trees of *A. marina* from New Zealand [19]. Increasing growth rates with increasing wood density in *A. marina* is largely due to its unique wood anatomy, which includes phloem and parenchyma tissues (which have low density) within the wood. High wood density in *A. marina* is associated with thick fibre walls and low levels of phloem as well as large vessel diameters, which facilitate water transport, and high rates of photosynthetic carbon gain, while, low-density wood has high amounts of phloem in wood and smaller vessels, traits that are evident in trees growing under higher salinity regimes [18], [19]. Therefore, relatively rapid growth rates are achieved with dense wood in *A. marina*.

Our linear regression analyses indicated that wood density decreased from pith to bark (Figure 3A) and with age (Figure 3B) in the *A. marina* mangroves trees from the Exmouth Gulf. Radial variation of wood density in trees can be due to both (1) variation in climate over time and (2) tree aging [53], [54], [55]. Variation in wood density is responsive to variation in climate because while growth occurs, wood anatomical characteristics (e.g. xylem vessel diameter and fibre wall thickness), which are the basis of wood density variation, vary to respond to changing environmental conditions. In mangroves, changes in wood anatomy have been found to be associated with the availability of freshwater (i.e. rainwater or groundwater) and variation in the salinity of the water in which mangroves occur [16], [56]. Reductions in wood density, given that low wood density is associated with low growth rates in *A. marina* (see above), strongly suggest declining growth rates of trees over time.

However, as trees age a pattern of decreased wood density towards the bark can occur because when a woody stem ages, the inner part of the stem (originally functional sapwood) is transformed to heartwood (which lacks functional xylem vessels and parenchyma). Heartwood is denser than sapwood and is formed by polymerization of compounds such as lignin and flavonoids [57], [58], although we did not observe this in our wood sections. Additionally, as canopy size usually increases with tree age, wood density is often observed to decrease because the size of xylem vessels increases to accommodate larger water fluxes through the stem [59]. However, increases in canopy size of *A. marina* would be expected to be associated with increasing wood density [19]. Detrended wood density (which does not incorporate tree age effect) was variable over time, but significantly decreased with increments in the PDO. The significant decreases in wood density in *A. marina* with increments in the PDO can be partly explained by deteriorating environmental conditions, e.g. increased soil salinity due to reduced rainfall, but wood density was not significantly correlated with rainfall, indicating that rainfall was not the only climatic factor determining wood density variability.

We tested sensitivity of growth rates to the PDO and rainfall, but our results were not significant. Therefore other factors in addition to rainfall, that may interact with variation in rainfall (e.g. variation in sea level pressure or temperature), may also be influencing mangrove growth in the region (Supplementary material Figure S4) [7]. Growth rates tended to be higher during the period between 1952 and 1976, which was an extended negative phase of the PDO, than growth rates from the extended positive PDO phase between 1977 and 1998 (Supplementary material Figure S3). However, the differences in growth rates were not significant (Table 2). Growth rate determinations are variable because of uncertainties associated with dating and sampling wood for dating and because of variation in the performance of individual stems that vary due to spatial variation in growth conditions in the forest. The variation in the relationships between growth and the PDO may also reflect the high variability in the rainfall and PDO (*r^2^* = 0.16) relationship (Figure 1A, B). Increased replication over time would assist in unraveling the effects of variation in rainfall, temperature and sea level on mangrove growth in the region [60].

## Conclusions

The Pacific Decadal Oscillation Index is a weak but long-term proxy of rainfall in the Exmouth Gulf, Western Australia. Small, 10 cm diameter seaward fringing stems of *A. marina* in the Exmouth Gulf, Western Australia are relatively old, 48±1 to 89±23 years old (mean ±1**σ**). The wood density of our studied mangrove trees decreased with increments in the Pacific Decadal Oscillation Index. Future predicted drying of the region will likely lead to further reductions in wood density and their associated growth rates in mangrove forests of the region.

## Supporting Information

Figure S1 **Wood density profiles displayed as distance from the pith from four stems collected in Giralia Bay, Western Australia.** A–D) Wood density profiles of the four stems (*stem 1* – *stem 4*) with their EPS value of 0.97. E–H) Wood density profiles showing arithmetic means ± 1**σ** of the four stems. I–L) Detrended wood density profiles depicting arithmetic means ± 1**σ** of the four stems.
(TIFF)

Figure S2 **Wood density profiles over time from four stems collected in Giralia Bay, Western Australia.** A–D) Wood density profiles of the four stems (*stem 1* – *stem 4*) with their EPS value of 0.92. E–H) Wood density profiles showing arithmetic means ± 1**σ** of the four stems. I–L) Detrended wood density profiles depicting arithmetic means ± 1**σ** of the four stems.
(TIFF)

Figure S3 **Pacific Decadal Oscillation Index.** Mean annual Pacific Decadal Oscillation Index (PDO) from 1940 – 2008.
(TIFF)

Figure S4 **Relationship between sea level pressure and the Pacific Decadal Oscillation Index.** Relationship between A) mean annual sea level pressure (dashed line) and the Pacific Decadal Oscillation Index (PDO, solid line) in the Exmouth Gulf, Western Australia between 1951 and 2008. B) The line represents the linear regression where Sea Level Pressure  = 0.44 PDO +9.98, *r^2^* = 0.32, *p*<0.0001, *n* = 58.
(TIFF)
