# Supplementary figures and images for: Radiocarbon Dating and Wood Density Chronologies of Mangrove Trees in Arid Western Australia
Source: PLoS One. 2013 Nov 12;8(11):e80116. doi: 10.1371/journal.pone.0080116 (PMC3827189; doi:10.1371/journal.pone.0080116)

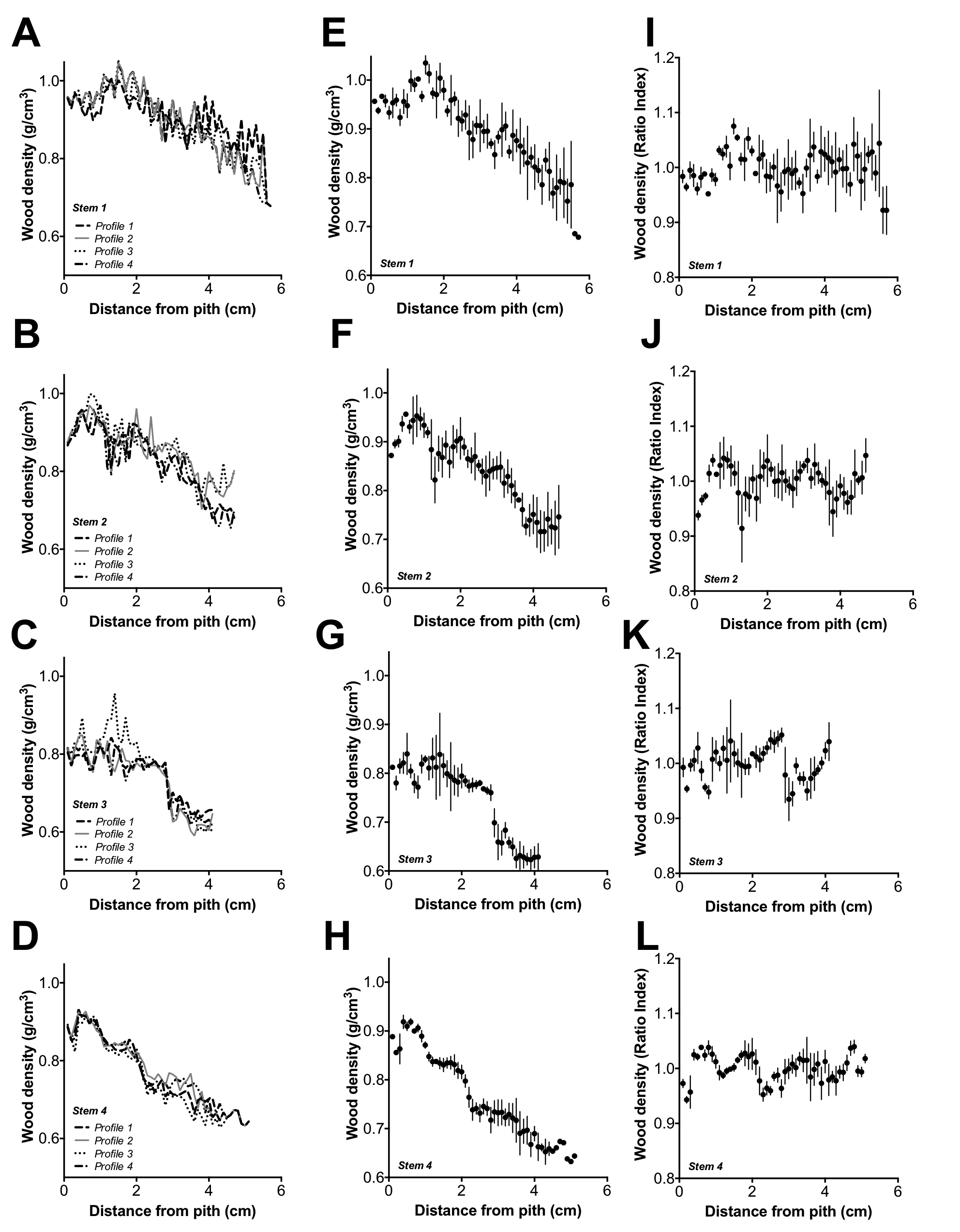

Supplement: Figure S1 — Wood density profiles displayed as distance from the pith from four stems collected in Giralia Bay, Western Australia. A–D) Wood density profiles of the four stems (stem 1 – stem 4) with their EPS value of 0.97. E–H) Wood density profiles showing arithmetic means ± 1σ of the four stems. I–L) Detrended wood density profiles depicting arithmetic means ± 1σ of the four stems. (TIFF) [file pone.0080116.s001.tiff]

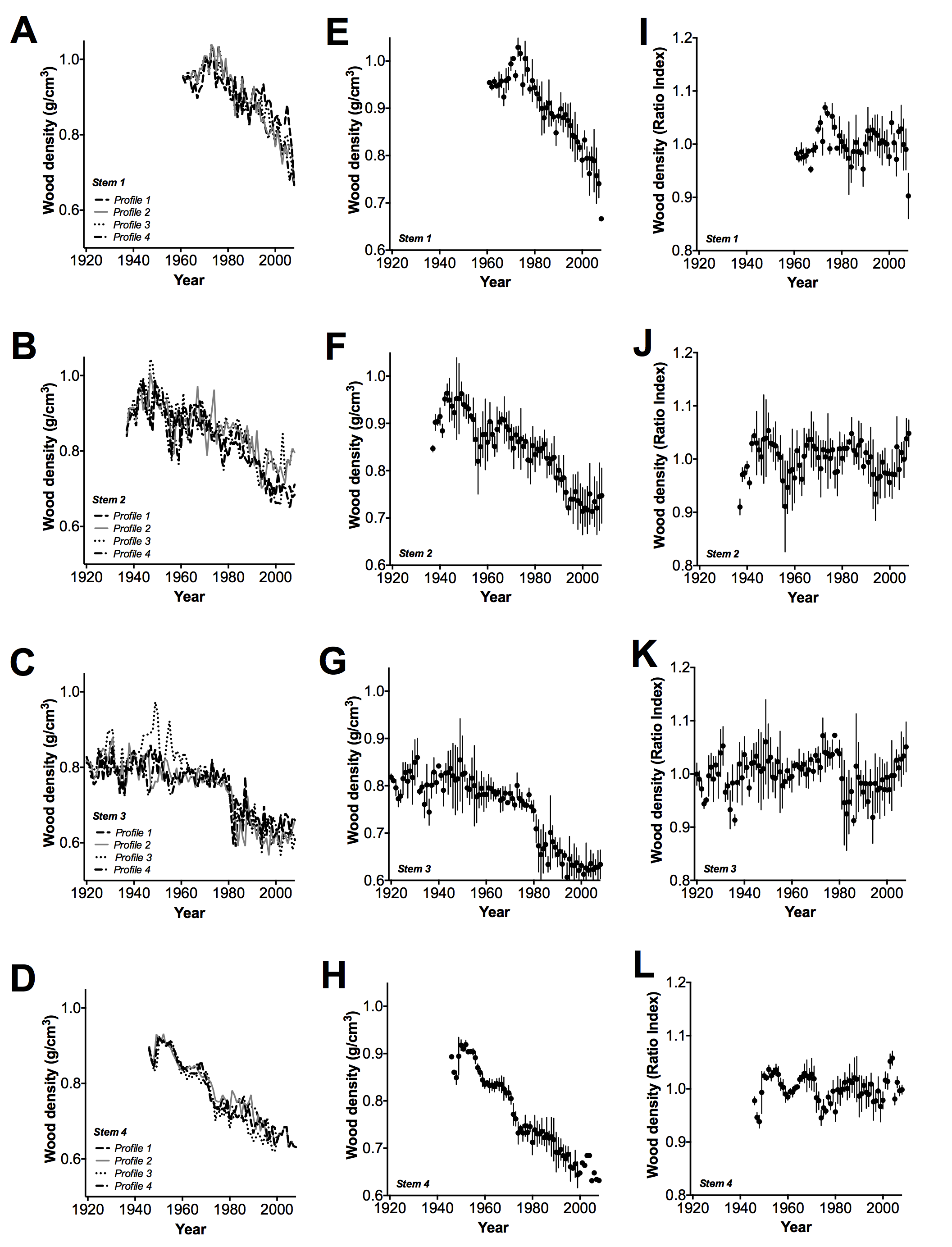

Supplement: Figure S2 — Wood density profiles over time from four stems collected in Giralia Bay, Western Australia. A–D) Wood density profiles of the four stems (stem 1 – stem 4) with their EPS value of 0.92. E–H) Wood density profiles showing arithmetic means ± 1σ of the four stems. I–L) Detrended wood density profiles depicting arithmetic means ± 1σ of the four stems. (TIFF) [file pone.0080116.s002.tiff]

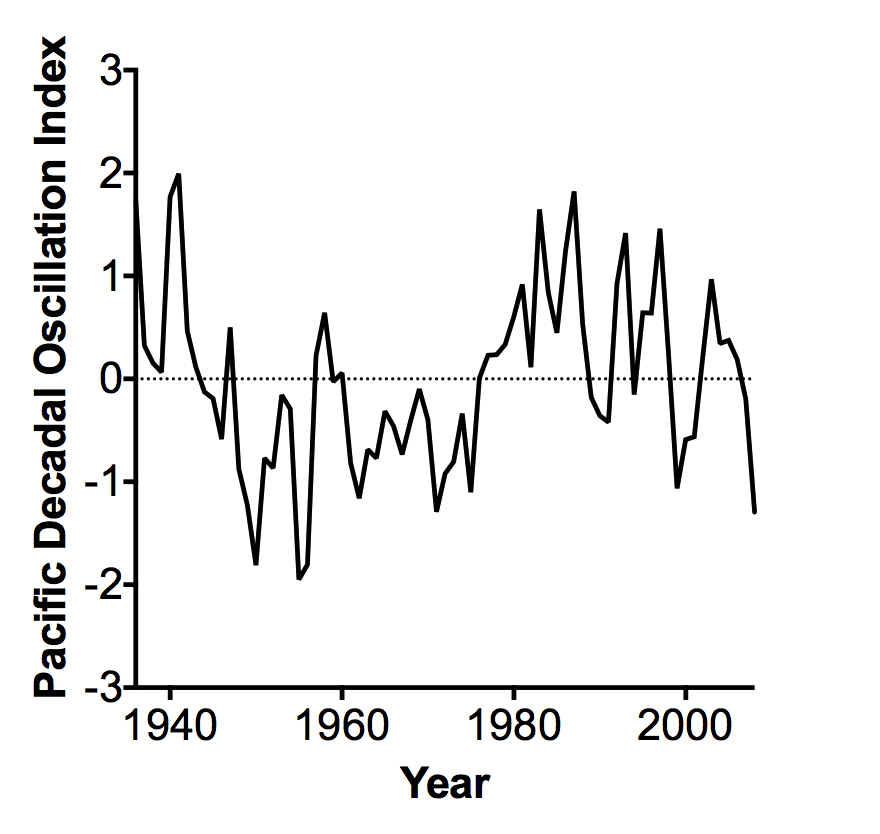

Supplement: Figure S3 — Pacific Decadal Oscillation Index. Mean annual Pacific Decadal Oscillation Index (PDO) from 1940 – 2008. (TIFF) [file pone.0080116.s003.tiff]

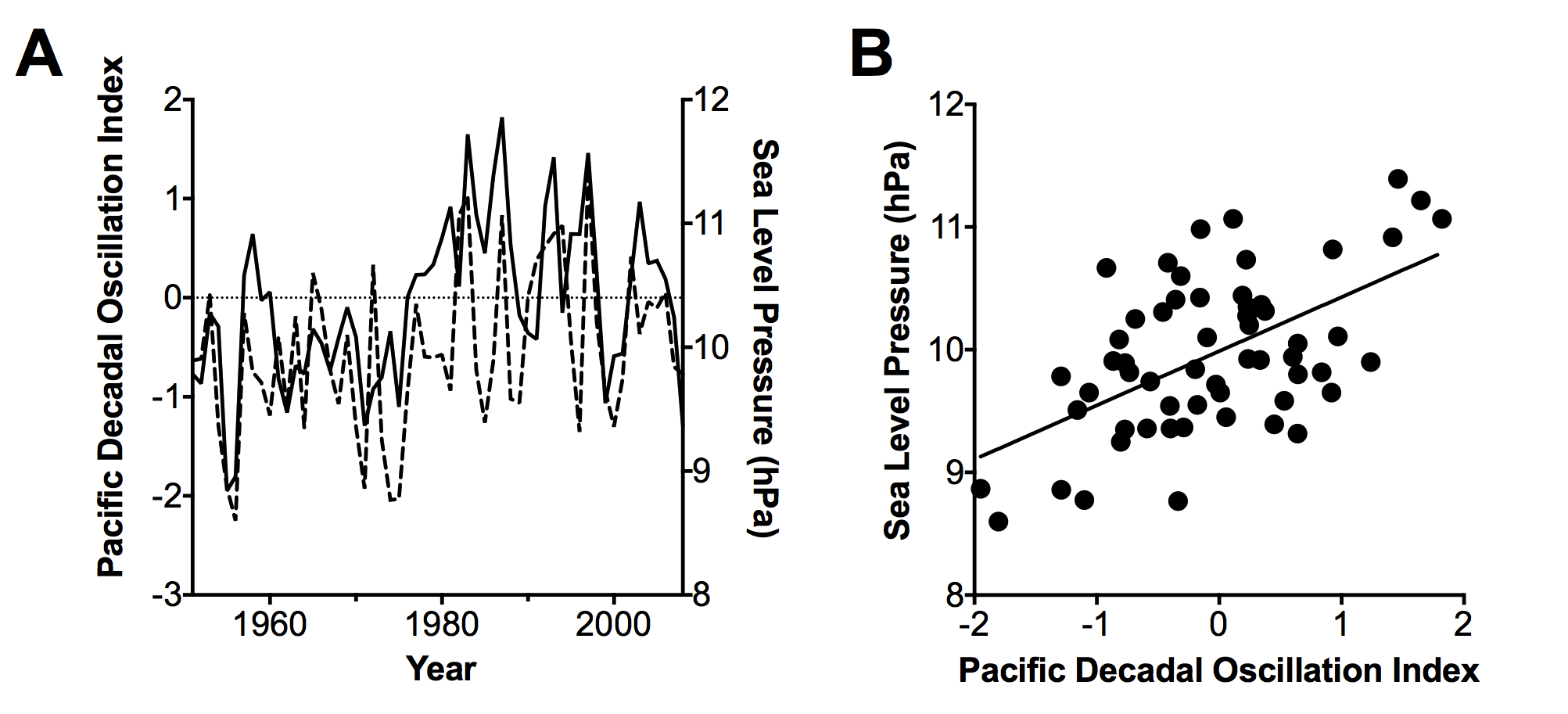

Supplement: Figure S4 — Relationship between sea level pressure and the Pacific Decadal Oscillation Index. Relationship between A) mean annual sea level pressure (dashed line) and the Pacific Decadal Oscillation Index (PDO, solid line) in the Exmouth Gulf, Western Australia between 1951 and 2008. B) The line represents the linear regression where Sea Level Pressure = 0.44 PDO +9.98, r2 = 0.32, p<0.0001, n = 58. (TIFF) [file pone.0080116.s004.tiff]
